# Food Bioactive HDAC Inhibitors in the Epigenetic Regulation of Heart Failure

**DOI:** 10.3390/nu10081120

**Published:** 2018-08-18

**Authors:** Levi W. Evans, Bradley S. Ferguson

**Affiliations:** 1 Department of Agriculture, Nutrition, & Veterinary Sciences, University of Nevada, Reno, NV 89557, USA; levi659@gmail.com; 2 Center for Cardiovascular Research, University of Nevada, Reno, NV 89557, USA; 3 Environmental Science & Health, University of Nevada, Reno, NV 89557, USA

**Keywords:** heart failure, histone deacetylase, HDAC, HDAC inhibitors, food bio-actives, phytochemicals

## Abstract

Approximately 5.7 million U.S. adults have been diagnosed with heart failure (HF). More concerning is that one in nine U.S. deaths included HF as a contributing cause. Current HF drugs (e.g., β-blockers, ACEi) target intracellular signaling cascades downstream of cell surface receptors to prevent cardiac pump dysfunction. However, these drugs fail to target other redundant intracellular signaling pathways and, therefore, limit drug efficacy. As such, it has been postulated that compounds designed to target shared downstream mediators of these signaling pathways would be more efficacious for the treatment of HF. Histone deacetylation has been linked as a key pathogenetic element for the development of HF. Lysine residues undergo diverse and reversible post-translational modifications that include acetylation and have historically been studied as epigenetic modifiers of histone tails within chromatin that provide an important mechanism for regulating gene expression. Of recent, bioactive compounds within our diet have been linked to the regulation of gene expression, in part, through regulation of the epi-genome. It has been reported that food bioactives regulate histone acetylation via direct regulation of writer (histone acetyl transferases, HATs) and eraser (histone deacetylases, HDACs) proteins. Therefore, bioactive food compounds offer unique therapeutic strategies as epigenetic modifiers of heart failure. This review will highlight food bio-actives as modifiers of histone deacetylase activity in the heart.

## 1. Introduction

Cardiovascular disease (CVD) remains the leading cause of death worldwide [1]. Moreover, CVD and its related co-morbidities financially strain the healthcare system in which total U.S. medical cost is estimated at $656 billion. Costs are expected to rise to $1.1 trillion by 2035 [1]. As a consequence, the American Heart Association (AHA) has initiated strategies aimed to reduce healthcare burdens that entail behavior modifications such as changes in dietary choices [1]. 

Heart failure (HF) is a cardiovascular condition in which the heart fails to deliver an adequate supply of oxygen-rich and nutrient-rich blood to the body [2]. Currently, 5.7 million U.S. adults are diagnosed with HF with a projected increase to 8 million of U.S. adults by 2030 [3]. Standards of care for the treatment of HF include angiotensin converting enzyme inhibitors (ACEi) and β-blockers [4]. Despite overall improvements in total HF mortality rates over the last several decades due to these therapies, five-year mortality rates post-HF diagnosis remain high at approximately 50% [3]. This further warrants behavioral dietary interventions or novel pharmaceuticals and/or nutraceuticals that effectively prevent and/or treat HF.

Multiple stressors including hypertension and inflammation stimulate the heart to undergo remodeling. Cardiac remodeling is characterized by heart enlargement (hypertrophy) and fibrosis (scarring) as well as contractile dysfunction and apoptosis [5]. All of these conditions can contribute to the progression of HF. Standard treatments such as ACEi and β-blockers target intracellular signaling cascades and disrupt cell surface receptors in order to inhibit cardiac remodeling and improve contractile function. For example, β-blockers act as competitive and reversible antagonists of β-adrenergic receptors (β-ARs). HF is associated with adrenergic nervous system hyper-activity that results in stimulation of β-ARs and leads to increased oxygen demand and myocardial work. β-AR hyper-activation ultimately contributes to increased intracellular signaling cascades that drives apoptotic signaling, cardiac enlargement, and cardiac contractile dysfunction. Thus, treatment with β-blockers attenuates these actions and improves systolic cardiac function [2,6,7,8]. However, inhibition of cell surface receptors and/or intracellular signaling cascades does not account for signaling cross-talk and redundancy, which limits the current therapeutics from completely inhibiting or reversing cardiac dysfunction. In other words, current therapies fail to inhibit all downstream regulators of cardiac disease. This has given rise to drugs that target the epi-genome. 

**Figure 1:**
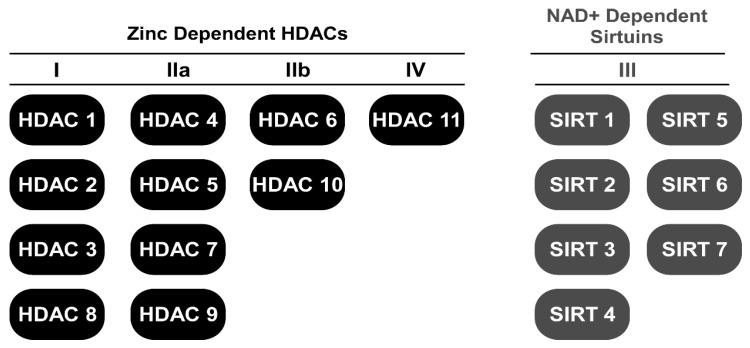
Schematic for HDAC classes dividing zinc-dependent HDACs from NAD^+^-dependent HDACs.

It has been reported that histone deacetylase (HDAC) activity is elevated in models of cardiac remodeling [9,10,11,12]. However, its activity in human heart failure, to our knowledge, has not been reported. Nonetheless, class I and II HDAC inhibitors represent a group of small molecule epigenetic modifiers that have demonstrated efficacy in animal models of HF over the last decade [11,13,14,15,16,17,18,19]. HDACs remove and histone acetyl transferases (HATs) add acetyl-marks to the ε-amino terminal tails of histones in nucleosomal DNA [20]. Deacetylation of histones via HDACs generally results in heterochromatin formation and gene repression while acetylation via HATs promotes gene expression [20]. Currently, 18 mammalian HDACs have been grouped into one of four classes (Figure 1): class I (HDAC1, 2, 3, and 8), class II (HDAC4, 5, 6, 7, 9, and 10), class III (SIRT1-7) and class IV (HDAC11). HDAC classes I, II, and IV require zinc as a cofactor to catalyze deacetylase activity while class III HDACs, which is also known as the sirtuins, require the cofactor nicotinamide adenine dinucleotide (NAD^+^). Class II HDACs are further subdivided into IIa (HDAC4, 5, 7, and 9) and IIb (HDAC6 and 10) [21]. Unlike class I and II HDACs, activation of class III HDACs (sirtuins) appears cardio-protective [22,23]. As such, a majority of this review will focus on the regulation of lysine acetylation via zinc-dependent HDACs.

## 2. HDAC Inhibitors

HDAC inhibitors were originally studied in cancer since different cancer cells expressed patterns of histone hypo-acetylation. Cancer cell hypo-acetylation has been associated with cancer progression. Treatment with HDAC inhibitors ameliorated cancer hypo-acetylation along with several hallmarks of cancer including proliferation and cancer cell survival [24,25]. Since these early studies, four HDAC inhibitors – Vorinostat, Romidepsin, Panobinostat, and Belinostat – have been approved by the US Food and Drug Administration (FDA) to treat T-cell lymphoma. At least 12 more HDAC inhibitors are in clinical trials for various cancers [26,27,28,29]. In addition, valproic acid, which is a short-chain fatty acid HDAC inhibitor, has been approved to manage epilepsy [30]. However, there are no HDAC inhibitors currently on the market or in clinical trials for the treatment of CVD/HF.

The classic zinc-dependent HDAC inhibitor structure is characterized by a cap, which is a zinc-binding domain within the active site and a hydrocarbon linker that connects the cap and binding domain [31,32,33]. Moreover, HDAC inhibitors have been categorized into five chemical classes known as hydroxamic acids, short-chain fatty acids, benzamides, ortho-aminoanilides, and cyclic peptides [33,34]. Differences amongst HDAC inhibitors include toxicity and potency [33,35]. For example, hydroxamic acids such as Vorinostat exhibit strong chelating properties that allow for pan-HDAC inhibition at nanomolar concentrations. Conversely, short-chain fatty acids such as valproic acid, exhibit weaker potencies with inhibition observed at milli-molar concentrations. In addition, while short-chain fatty acids elicit physiochemical properties that allow for easy uptake and transportation, they lack specificity and, therefore, have multiple off-target actions [30,33]. Benzamines and ortho-aminoanilides are structurally similar and are often selective of class I HDACs. Lastly, cyclic peptides such as Romidepsin are characterized by many alkyl-binding and chelating-binding properties that permit their high potency [36]. This review will primarily discuss class I and II HDACs and HDAC inhibitors. 

## 3. HDAC Inhibitors and Heart Failure

The role for HDACs in the heart have been researched for over a decade. Mechanisms and functions of HDACs in the heart are complex and actions differ between HDAC classes and experimental techniques as well as genetic versus pharmacological inhibition. For example, results from *in vitro* and *in vivo* experiments have suggested that class IIa and III HDACs are cardio-protective where pharmacological or genetic inhibition contributes to cardiac dysfunction [22,37,38]. Classical genetic loss-of-function studies demonstrated that class IIa HDACs bind the transcription factor myocyte enhancer factor-2 (MEF-2) that resulted in transcriptional repression of hypertrophic genes. Knockout of class IIa HDACs, HDAC4 and 5, resulted in MEF-2 transcriptional activation and dilated cardiomyopathy [10,38,39]. These studies ultimately demonstrated that in response to stress, calcium-mediated activation of calmodulin-dependent protein kinase (CaMK) stimulated the dissociation of class IIa HDACs from MEF2, which resulted in MEF2 activation and pathological cardiac hypertrophy [40]. 

Like class IIa HDACs, early loss-of-function studies suggested a critical developmental role for class I HDACs where whole animal knockout of HDACs 1, 2 or 3 was shown to be embryonic or perinatal lethal [11,41,42,43]. Cardiac-specific knockout studies of HDACs 1, 2 and 3 was also lethal in a TAC-induced model of heart failure with lethality observed in rodents at postnatal day 14 [11]. In contrast to class IIa HDACs, however, small-interfering RNA-mediated knockdown of class I HDACs attenuated cardiac hypertrophy in cell culture [19,44]. Since these early studies, class I HDAC activity has been further observed to increase with cardiac remodeling and dysfunction [12,45,46]. These observations suggest multiple actions for class I HDACs in addition to their deacetylase function. 

Not surprising then, pan- and class I-selective HDAC inhibitors are efficacious in pre-clinical models of HF. Trichostatin A (TSA), for example, is a pan-HDAC inhibitor that has been shown to inhibit pathological cardiac hypertrophy and fibrosis [47]. While TSA has been shown to regulate histone hyper-acetylation and gene expression [48,49], its actions on pathological heart enlargement appear to be regulated, in part, through inhibition of mitogen-activated protein kinase (MAPK) signaling [50]. These data would suggest epigenetic and non-epigenetic (e.g., signaling mediated) mechanisms of action. Similar results were shown when treated with class I-selective HDAC inhibitors in which cardiac hypertrophy and fibrosis were attenuated [19,50,51]. It should be noted that differences between the class I HDACs, HDACs 1 and 2 can be difficult to distinguish with pharmacological tools. This is due to the high sequence homology between the two HDACs and their redundant actions toward histone targets. The use of genetic and pharmacological tools suggest that inhibition of HDACs 1/2, HDAC3 or HDAC8 in combination or individually attenuated cardiac remodeling and improved cardiac function [19,46,50,52,53]. Therefore, class I-selective HDAC inhibition as opposed to pan-HDAC inhibition may offer better therapeutic strategies with limited off-target consequences.

Like the class I HDACs, class IIb HDAC activity is increased in the heart in models of hypertension [12]. Moreover, genetic or pharmacological inhibition of the class IIb HDAC, HDAC6, improved systolic contractile function independent of cardiac enlargement and fibrosis in a rodent model of hypertension [54]. Similarly, genetic or pharmacological inhibition of HDAC6 was reported to ameliorate cardiac proteotoxicity by preventing protein aggregation through improved autophagy-mediated protein degradation [55]. Unlike class I HDACs, HDAC6-mediated regulation in these studies was directed at sarcomere protein deacetylation [54] or tubulin hyperacetylation [55], which suggests that the class IIb HDAC, HDAC6 regulates cardiac function through non-epigenetic mechanisms. 

Lastly, the most recent studies have shown that the FDA-approved HDAC inhibitor Vorinostat as well as Givinostat (ITF2357), which is currently in phase III clinical trials for patients with Duchenne muscular dystrophy, attenuated and even reversed cardiac dysfunction in rabbits exposed to ischemia reperfusion (I/R) injury [16] and in aged mice with diastolic failure [56]. These reports highlight the efficacy of HDAC inhibitors for treating and potential reversing cardiac disease. In addition, these reports relied on HDAC inhibitors that are currently FDA approved or undergoing human clinical trials. 

Unfortunately, many identified HDAC inhibitors are expensive to synthesize and are not likely to see human HF trials due to their off-target effects [57,58]. Conversely, nutraceutical phytochemicals provide a cheaper and safer alternative to pharmaceuticals. It was recently delineated that HDAC inhibitors have a common phenyl ring that governs their biological activity [59]. These findings are interesting since multiple phytochemicals in our foods have phenyl rings that drive their bioactivity. This suggests that the chemicals in our foods may improve health via acetyl-lysine modification in addition to their well-established roles in oxidative stress and inflammation. 

## 4. Phytochemicals

Diet and nutrition play a key role in health and disease in which dietary intervention can ameliorate type II diabetes, cancer progression, and CVD [60]. Poor dietary habits attribute 13.2% to overall CVD mortality in the U.S. [1]. Similarly, hyper-caloric intake is linked to the development of hypertension and type II diabetes, which are two major risk factors for CVD and HF [61]. The American Heart Association, the World Health Organization, and the Academy of Nutrition and Dietetics have stressed that the consumption of fruits, vegetables, and other plant-based foods should compose the majority of one’s diet to reduce the risk of developing CVD and other morbidities [62,63,64,65,66]. These foods are high in vitamins, minerals, and phytochemicals that actively participate in biological processes that govern health. This is evident by the lower mortality rates for HF patients on the dietary approaches to stop hypertension (DASH) diet or the Mediterranean diet; these diets emphasize plant-based foods [67]. Unfortunately, plant-based foods that contain beneficial nutrients and phytochemicals are, for the most part, under-consumed in the U.S. [1,62].

Phytochemicals are secondary plant metabolites that are synthesized to help a plant thrive or deter competitors, predators and pathogens [68,69]. Phytochemicals can further interact in human biological processes after ingestion to promote health. Fruits, vegetables, nuts, seeds, legumes, whole grains, herbs and natural spices are common dietary items that contain phytochemicals in varying concentrations. Moreover, phytochemicals and their parent plants have been used in traditional medicines for centuries. Thousands of phytochemicals have been identified to date with more that are likely to be discovered and characterized [70]. Currently, phytochemicals are characterized into one of six different classes: polyphenols/phenolics, alkaloids, N-containing compounds, organosulfur compounds, phytoesterols and carotenoids [71]. Following is a brief description of the different phytochemical groups as well as compounds within these groups that regulate lysine acetylation and their implications in HF. A list of these compounds and their respective roles in the regulation of HDAC activity and histone acetylation can be found in Table 1. 

### 4.1. Polyphenols

The structure of polyphenols have been intensively reviewed [105,106,107]. Polyphenols are highly abundant in the plant kingdom and comprise a family of molecules with more than 8000 structural variants. These secondary metabolites contain many aromatic rings with one or more hydroxyl moieties [108]. Hydroxyl groups are classically recognized in oxidation-reduction reactions. Thus, many studies have focused on the anti-oxidant role for polyphenols in CVD [68]. Since polyphenols are among the most abundant bioactive molecules in the plant kingdom, it is not surprising that polyphenols are among the most abundant phytochemicals consumed in the human diet. For this reason, polyphenols are important compounds to study in human health and disease. While oxidative stress and inflammation are the classical targets for polyphenol health protection, recent research indicates an important role for polyphenols in diet-gene regulation [109,110]. 

Polyphenols are divided by chemical structure into two primary groups: phenolic acids and flavonoids. Moreover, polyphenols are distinguished by their hydroxyl moiety and their aromatic phenyl rings. Phenolic acids contain the subgroups hydroxycinnamic acids and hydroxybenzoic acids while flavonoids contain the subgroups flavanols, flavonols, flavones, flavanones, anthocyanidins, isoflavonoids and proanthocyanidins. Other polyphenol groups include lignans, stilbenes, and quinones. Below, we highlight the role for these polyphenol subgroups and their compounds as epigenetic regulators in the heart.

#### 4.1.1. Phenolic Acids

Studies suggest that phenolic acids are inversely correlated with coronary heart disease mortality and heart attack incidence [111]. Phenolic acids contain two subgroups including hydroxycinnamic acids and hydroxybenzoic acids, which differ in carbon backbone length. Hydroxycinnamic acids contain an additional carbon bond. Both hydroxycinnamic acids and hydroxybenzoic acids contain a functional carboxyl group with potent metal chelation properties [112]. This would imply that hydroxycinnamic acids and hydroxybenzoic acids can chelate zinc in order to inhibit zinc-dependent HDAC activity. Docking studies using HDAC8 confer that the carboxylic group of phenolic acids strongly interacts with the zinc ion, which results in high HDAC inhibition potency [112]. Below, we discuss recent findings regarding phenolic acid HDAC inhibitors in the heart. 

#### 4.1.2. Hydroxycinnamic Acids

Caffeic acid is one of the most abundantly consumed hydroxycinnamic acids [72]. Caffeic acid is found in most fruits including the skin of ripened fruit [113]. However, the largest source for caffeic acid consumption is coffee [114]. Coffee has been linked to improvements in CVD where coffee consumption was inversely correlated with death after acute myocardial infarction [73]. These epidemiological findings suggest that coffee and its phytochemicals have cardio-protective effects. Unfortunately, the emergence of energy drinks has given rise to misinformation regarding coffee consumption and arrhythmias. Very high doses of caffeine have been reported to have sympathomimetic effects likely caused by phosphodiesterase inhibition and increased intracellular calcium. In this regard, energy drinks have been linked in numerous case reports with atrial and ventricular tachyarrhythmias. However, coffee consumption of three cups per day did not increase the risk of atrial fibrillation or ventricular arrhythmias [115]. Further *in vitro* and *in vivo* reports demonstrated efficacy for caffeic acid in CVD models [116]. Caffeic acid ethanolamide, which is a caffeic acid derivative, ameliorated cardiac oxidative stress in isoproterenol-induced HL-1 cells as well as in isoproterenol-induced cardiac diseased mice [116]. Additionally, caffeic acid attenuated cardiac dysfunction and fibrosis through HDAC regulation [116]. Similar to the pan-HDACi Vorinostat, caffeic acid phenethyl ester attenuated cardiac hypertrophy and ameliorated cardiac dysfunction in I/R-injured rabbits [117]. These therapeutic actions occurred, in part, by inhibiting MAPK activation [118]. Since HDACs have been shown to regulate MAPK activity [50], these data suggest that caffeic acid-mediated inhibition of HDACs protect the heart via MAPK inactivation. More recently, caffeic acid was shown to inhibit class I, IIa, and IIb HDAC activity in cardiac lysate [119]. Unfortunately, no other studies have further examined the role for caffeic acid as a zinc-dependent HDAC inhibitor in heart failure. Further delineation of the cardio-protective actions of caffeic acid and its derivatives would be of great interest due to their high intake through coffee consumption. Additionally, other dietary hydroxycinnamic acids such as coumaric acid and ferulic acid should be examined as regulators of HDAC activity in the heart. Both coumaric acid and ferulic acid have been reported to attenuate pathological cardiac remodeling. In addition, studies suggest that ferulic acid inhibits HDAC activity [74,120,121,122,123]. Combined, these studies would suggest that hydroxycinnamic acids protect the heart, in part, through direct changes in gene expression. Hydroxycinnamic acids inhibit HDAC activity, which leads to hyper-acetylation of nucleosomal histones.

#### 4.1.3. Hydroxybenzoic Acids

Compared to other phenolic acids, hydroxybenzoic acids are consumed less and have lower phytochemical concentrations within food [124]. However, berries such as blackberries and strawberries are commonly consumed and contain substantial amounts of the hydroxybenzoic acids known as gallic acid and ellagic acid. Black tea is also a good source of gallic acid and is of particular interest due to its large consumption and its correlation with reduced risk for coronary heart disease as well as stroke [125,126]. In addition, these compounds have been examined as nutraceuticals that can protect the heart [127,128,129,130]. For example, gallic acid has been shown to repress cardiac remodeling through the inhibition of genes involved in advanced glycation end products (AGE) in rats [127]. Moreover, Umadevi et al. [127] reported that gallic acid attenuated cardiac fibrosis by inhibiting matrix melloproteinase (MMP) gene expression of MMP-2 and MMP-9. Inhibition of MMP gene expression was linked to decreased inflammation and intracellular signaling cascades nuclear factor kappa B (NF-κB) and extracellular signal-regulated kinase (ERK). HDACs have been reported to regulate both NF-κB and ERK signaling where HDAC inhibition attenuated NF-κB and ERK activity [50,51,131]. These data suggest that cardio-protective actions of gallic acid are partially mediated through HDAC inhibition. Gallic acid was shown to dose-dependently inhibit class IIa and IIb HDAC activity, which resulted in cardiac protection [128]. While this study supports that hydroxybenzoic acid HDAC inhibitors protect the heart through changes in gene expression, the evidence is far from conclusive. Thus, further studies are warranted to examine the role for gallic acid and other hydroxybenzoic acids on global changes in histone acetylation and gene expression.

#### 4.1.4. Flavonoids

The largest polyphenolic group known as flavonoids are aglycone structures that contain two active phenyl rings, which vary in hydroxylation between its subgroups: flavanols, flavonols, flavones, flavanones, anthocyanidins, isoflavonoids and proanthocyanidins. Currently, there are approximately 6000 flavonoids that are found in fruits, vegetables, herbs and medicinal plants. Research has shown that diets high in flavonoids reduced a person’s risk for developing CVD as well as reduced CVD mortality rates [132,133]. Moreover, a meta-analysis of 15 cohort studies with 386,610 individuals and 16,693 deaths showed flavonoid intake was inversely correlated with CVD mortality in a dose-dependent manner [134]. Such findings confirm the importance of and validate policies directed towards consuming more fruits and vegetables. Notably, reports have shown that flavonoids have metal-binding chelating properties [135,136] and, therefore, suggest potential roles for flavonoids as HDAC inhibitors for cardio-protection.

#### 4.1.5. Flavonols

Flavonols are 3-hydroxy derivatives of flavones and contain a number of commonly studied phytochemicals that include quercetin. Quercetin is the most consumed flavonol and is abundant in tea, apples, onions and berries [137,138]. Quercetin intake is inversely correlated with ischemic heart disease mortality in a dose-dependent manner [139]. In addition, quercetin has been shown to protect against ischemia/reperfusion injury, isoproterenol-induced cardiac injury, aortic constriction-induced cardiac remodeling and diabetic cardiomyopathy [75,76,77,140,141]. Two independent double-blind, placebo-controlled trials demonstrated that quercetin ameliorated hypertension in patients at risk for CVD and reduced plasma oxidized low-density lipoproteins (oxLDLs), which are responsible for atherosclerotic disease [142,143]. Few reports, however, have shown quercetin’s mechanistic action of cardio-protection through acetyl-lysine regulation. Hung et al. showed that quercetin attenuated oxLDL-induced atherosclerotic injury by increasing the class III HDAC Sirt-1 [144]. Our lab demonstrated that quercetin inhibited class I and II HDACs in bovine cardiac tissue [119]. Other studies have reported that quercetin can inhibit class I HDACs in cancer cell models and that these actions are, in part, responsible for the anti-carcinogenic actions associated with quercetin [145,146]. As an HDAC inhibitor, quercetin would alter the electrostatic interactions between DNA and histone proteins, which is directly impacting gene expression and, therefore, effecting cellular fate. While the role for quercetin in cardio-protection is undeniable, studies examining the epigenetic impact for quercetin remain underexplored. Thus, further investigation for quercetin as an HDAC inhibitor in cardiac biology is warranted. 

Kaempferol is a flavonol found in a variety of foods like teas, tomatoes, hops, grapes, grapefruit, strawberries, broccoli, honey, apples and beans [147]. Kaempferol is the second-most consumed flavonol in the U.S. behind quercetin and is mostly consumed in the form of green and black tea [137]. Similar to quercetin, kaempferol intake is inversely correlated with ischemic heart disease mortality [139] and kaempferol treatment is efficacious in *in vitro* and *in vivo* CVD models [78,79,88,148,149]. I/R-induced cardiac injury was ameliorated with kaempferol treatment. This was linked to the inhibition of the MAPK pathway [79,148]. Since HDAC inhibitors have previously been shown to attenuate MAPK signaling in the heart, these data would suggest a potential role for kaempferol as an HDAC inhibitor [50,51]. Kaempferol has also been shown to attenuate cardiac injury and oxidative stress in I/R-injured rats by inhibiting glycogen synthase kinase-3β activation (GSK-3β) [149]. The class I HDAC, HDAC2 was recently shown to regulate GSK-3β signaling [150]. These data support the postulate that kaempferol protects the heart in an HDAC-dependent manner. Consistent with this postulate, kaempferol was recently shown to inhibit HDAC activity, which led to increased histone acetylation [151]. Berger et al. [151] further showed that kaempferol docked to class I HDACs 2 and 8 as well as class IIa HDACs 4 and 7, which suggests that this binding may inhibit HDAC activity. Lastly, we reported that kaempferol inhibited HDAC activity and increased histone acetylation in cardiac lysate [119]. As the next step, experiments are underway to determine if the cardio-protective effects of kaempferol are mediated through HDAC-dependent inhibition. These studies would also examine the impact for green and black tea extracts in regulating HDAC inhibition and cardiac disease even though additional tea compounds would likely impart additive or synergistic actions towards HDAC activity (e.g., EGCG). As others have shown that anti-carcinogenic actions for kaempferol are regulated, in part, through changes in lysine acetylation [152], we anticipate promising findings that would demonstrate that kaempferol-dependent HDAC regulation links diet-gene interactions in an epigenetic-dependent manner in the heart. 

Myricitrin and its aglycone, myricetin, are two naturally occurring flavonols that were first isolated in the early 1900s from the bark of the bayberry tree (Myrica nagi) [153]. Bayberry has been a cultural staple in Asian countries for over 2000 years [154] and the tree’s therapeutic properties in traditional medicines have led to current studies of these two flavonols. Myricitrin is primarily synthesized in the bayberry tree’s fruit, bark and leaves [155] while myricetin is also found in a variety of other foods including tea, wine, berries and vegetables. The majority of myricetin consumption is from tea. However, its intake is quite low in comparison to other flavonoids like kaempferol and quercetin [137]. The bioactivity of myricetin and myricitrin are very similar to each other due to the sharing of functional groups. Both phytochemicals exhibit anti-inflammatory and anti-oxidant properties [154,155], which have been suggested as a major mechanism for their cardioprotective actions [156,157,158]. However, additional studies have reported that cardio-protection for myricitrin and myricetin involve regulation of intracellular signaling cascades and gene expression. For instance, myricetin was shown to attenuate I/R-induced cardiac injury by inhibiting signal transducer and activator of transcription 1 (STAT1) activation [159]. Inhibition of JAK/STAT signaling would be expected to alter gene expression in the heart. Two other reports showed that myricitrin attenuated diabetic cardiomyopathy as well as hyperglycemia-induced cardiomyocyte apoptosis through changes in PI3K/Akt and MAPK signaling [160,161]. Cardiac myocytes exposed to hyperglycemic conditions and treated with myricitrin had reduced apoptosis via Akt-nuclear factor erythroid 2-related factor 2 (Nrf2) inhibition [160]. Similarly, myricitrin attenuated diabetic cardiomyopathy by inhibiting ERK phosphorylation, Nrf2 expression and NF-κB [161]. Since Nrf2 and NF-κB are transcription factors, these data would suggest that myricitrin regulates cardiac gene expression through the regulation of intracellular signaling cascades. HDAC inhibitors have previously been shown to regulate Akt [162], MAPK phosphorylation [50,51] and NF-κB [131]. Only one report to date, however, has shown myricetin and myricitrin regulated lysine acetylation through HDAC inhibition [119]. Thus, investigation into the role for these two compounds as bioactive HDAC inhibitors in the heart is warranted.

#### 4.1.6. Flavones

Flavones are synthesized from flavanones via flavone synthases. These polyphenols distinctly contain a double bond between carbons two and three on the heterocyclic pyran ring (also known as the C ring), which is further attached to an aromatic phenyl ring [163]. Multiple hydroxyl groups that are attached to this phenyl ring provide flavones with their function especially with regard to redox reactions [163]. Flavone consumption is less than flavonols, but these are well-represented in research studies. Apigenin and luteolin, as well as their glycosides, are two of the major flavones currently being investigated in the heart. Apigenin is found in citrus fruits, onions, parsley and chamomile [80]. Several reports have shown that apigenin is cardio-protective [81,164,165,166]. Similar to other flavonoids, apigenin was shown to attenuate I/R-induced cardiac injury by inhibiting MAPK signaling [81,165] and Nrf2 transcriptional activation [164]. These reports are interesting since they suggest that apigenin protects the heart through intracellular signaling and gene expression. Again, inhibition of HDACs has been linked to MAPK inactivation and control of the transcription factor activation [50,51,131]. In addition, we and others have shown that apigenin inhibits class I HDAC activity [119]. Inhibition of HDAC activity by apigenin has been linked to hyper-acetylation of histone proteins in cancer models that contributes to cancer cell death [167,168]. Collectively, these data would suggest that cardio-protective actions for apigenin is controlled, in part, via HDAC-dependent mechanisms that necessitate epi-genome wide changes in gene expression.

Luteolin is commonly found in celery, parsley, broccoli, onions, carrots, peppers, cabbages and apples [169]. These foods and other plants such as the chrysanthemum flower have been used in traditional Chinese medicine for the treatment of hypertension as well as for treating microbial infections [170]. Unlike other flavonoids, epidemiological studies examining the cardio-protective role for luteolin remains unclear [171,172]. This may partly be explained by the low intake of this flavone in the diet [139]. In the cell culture and rodent models, however, luteolin has shown clear cardio-protection. Mechanistic actions for luteolin generally involve the regulation of sarcoplasmic reticulum Ca^2+^-ATPase 2a (SERCA2a) [82,173,174]. SERCA2a is decreased in the failing heart, which leads to impaired calcium reuptake and cardiac contractile dysfunction [175]. Post-translational modification of SERCA2a has been suggested as critical for SERCA2a function. Modifications from small ubiquitin-related modifier 1 (Sumo1) and phosphorylation via MAPK activation appear vital for SERCA2a-dependent calcium re-uptake into the sarcoplasmic reticulum [175,176]. Recent findings showed that class I HDAC inhibition promoted SERCA2a SUMOylation [177]. This would be expected to improve cardiac contractility. Notably, luteolin was reported to inhibit class I HDAC activity as well as increase lysine acetylation on histone H3 in cardiac myoblasts [119]. Furthermore, docking studies demonstrated that luteolin binds within the catalytic domain of class I HDACs to inhibit HDAC activity [178]. Lastly, luteolin was reported to attenuate cardiac dysfunction by regulating Akt and MAPK signaling [174,179]. Similar to other flavonoids, these data would suggest that luteolin attenuates MAPK phosphorylation by inhibiting HDAC activity and the data suggest that this attenuates cardiac remodeling and dysfunction. This postulate is currently being tested. 

*Scutellaria baicalensis* was used as an herbal remedy in traditional medicine to treat bacterial and viral infections especially hepatitis, but it has since shown efficacy for the treatment of hypertension, inflammation, oxidative stress and cancer [180]. While over 50 flavonoids have been isolated from this mint plant for traditional Chinese and Japanese medicine, baicalin and baicalein constitute its major phytochemicals [181]. These two phytochemicals only differ in that baicalein has a distinguishable aglycone [182]. With regard to the heart, baicalein [183,184,185] and baicalin [83,84,85,186,187,188,189] have shown efficacy in ischemia-induced and isoproterenol-induced cardiac dysfunction. Similar to other flavonoids, baicalein and baicalin elicit cardio-protection by inhibiting oxidative stress and inflammation as well as attenuating MAPK signaling [178,179,180,183,185,186,187,188]. Baicalein was also reported to inhibit cardiac hypertrophy and fibrosis in mice exposed to aortic constriction [190]. This was partly explained by the inhibition of ERK phosphorylation [190]. Similar results were shown for baicalin in which baicalin-mediated ERK inactivation improved isoproterenol-induced cardiac dysfunction [188], bleomycin-induced pulmonary hypertension [84] and myocardial infarction [189]. These studies did not examine the epigenetic actions for baicalein or baicalin in regulating heart function. However, it has been reported that baicalein can inhibit HDAC4 and HDAC5 [191] while baicalin was shown to inhibit HDAC2 [192] and HDAC1 [193] in various models of disease. These findings demonstrate that baicalein and baicalin act as HDAC inhibitors. Coupled with our more recent findings that baicalein and baicalin inhibited HDAC activity in cardiac tissue [119], these data would suggest that future studies for these two phytochemicals as epigenetic regulators of cardiac function is warranted. 

#### 4.1.7. Flavanols 

Flavanols or catechins are structurally similar to flavonols but differ in the heterocyclic C ring. Flavanols do not contain a double carbon bond that allows four diastereoisomers to form from two chiral centers [194]. These phytochemicals are commonly found in chocolate, in the skins of apples and berries as well as in teas. Notably, epigallocatechin gallate (EGCG) is a flavanol that is abundant in the leaves of the green *Camellia sinensis* plant [195]. The compounds in these leaves are mostly consumed as the beverage green tea and have been used in traditional medicines for thousands of years around the world. Epidemiological research suggests that tea consumption is cardio-protective particularly in overweight and obese individuals [196]. Heart function improvements have been linked with the anti-oxidant and anti-inflammatory actions of EGCG, which are attributed to the eight hydroxyl groups on EGCG [86,87,197,198,199,200,201,202]. In these reports, EGCG was shown to inhibit diabetic cardiac dysfunction [198,199] and chemotherapy-induced cardiotoxicity [86,200]. In addition to its actions as an antioxidant and as anti-inflammatory, EGCG acts as a chelator [203,204]. This suggests that EGCG can interact with and chelate zinc within the catalytic domain of HDACs. In support of this, EGCG has been reported to inhibit HDAC activity even though docking studies have yet to be performed [119]. In addition, EGCG was shown to attenuate age-related cardiac dysfunction, in part, through increased acetylation of histone H3 at the cardiac troponin I promoter. This increased troponin’s expression and improved muscle function [205]. Increased histone acetylation was likely due to the inhibition of class I HDAC activity [205]. Additional reports have shown that EGCG inhibited HDAC3 activity in the heart, which also led to FoxO1 hyper-acetylation and attenuation of hyperglycemia-induced apoptosis [206]. FoxO1 plays an important role in apoptosis [9]. Based on these findings and considering that tea is heavily consumed worldwide, it would be interesting to elucidate HDAC activity in the peripheral blood mononuclear cells (PBMCs) of patients before and after green tea consumption. PBMCs have been used as indirect read-outs for disease states in patients with type II diabetes and CVD [207,208].

#### 4.1.8. Flavanonols

Flavanonols are 3-hydroxy derivatives of flavanones and are also known as dihydroflavonols [194]. Phytochemicals identified as flavanonols are sparse within the literature. However, dihydromyricetin is a flavanonol that has been implicated in health and disease [209]. With regard to the heart, reports have shown that dihydromyricetin is protective in I/R-induced cardiac injury [210], angiotensin II-induced cardiac fibrosis [211,212], diabetic cardiomyopathy [213] and lipopolysaccharide (LPS)-induced cardiac injury [214]. Dihydromyricetin elicited its cardio-protective effects, in part, by acting as an anti-oxidant, anti-inflammatory, and an inhibitor of the NF-κB pathway [211,212,213,214]. While no study has examined the role for dihydromyricetin in the epigenetic regulation of gene expression, recent findings from our lab showed that dihydromyricetin inhibited HDAC activity [119]. These data, while preliminary, highlight the potential for dihydromyricetin as an epigenetic modifier of gene expression for the prevention and or treatment of cardiac disease. 

#### 4.1.9. Proanthocyanidins 

Proanthocyanidins are abundant in the diet since they are found in fruits such as grapes, peaches, apples, pears and berries as well as wine, tea and beer [215]. These compounds are the subsequent products of catechins and form dimer, oligomer, and polymer complexes that promote their bioactivity [216]. Studies show that proanthocyanidins protect the heart in models of atherosclerosis. Many of these studies reported anti-oxidant and anti-inflammatory properties for proanthocyanidins [216]. For example, grape seed procyanidin (GSP) was shown to improve cardiac function by inhibiting inflammation and oxidative damage [217,218]. A systematic review/meta-analysis examined GSP intake in regulating blood pressure, heart rate, low density lipoprotein (LDL), high density lipoprotein (HDL) cholesterol, total cholesterol, triglycerides and C-reactive proteins [219]. This report demonstrated that GSP extract lowered systolic blood pressure and heart rate but did not significantly affect other cardiac markers. Other reports have shown that proanthocyanidins are efficacious for treating human hypertension [220]. Consistent with these reports, experimental rodent models of cardiac disease demonstrated that GSP extract protected the heart in response to a high fat diet [217,218,221], doxorubicin-induced cardiotoxicity [222,223,224,225,226], heavy metal-induced cardiac stress [227,228,229], isoproterenol-induced HF [230,231,232] and I/R injury [233,234,235,236,237]. Additional studies reported that GSP extract lowered liver and blood cholesterol as well as triglycerides [238,239,240,241]. This would suggest CVD protection. Moreover, GSP extract was shown to inhibit HDAC activity, specifically HDACs 2 and 3, and increase histone acetylation in the liver [241]. This was suggested to impact nuclear hormone receptor expression and lower serum triglycerides [241]. These results are interesting and suggests that cardio-protective actions of GSP result from HDAC inhibition. This postulate is currently under investigation. 

#### 4.1.10. Quinones

Plants contain enzymes including polyphenol oxidase that catalyzes a multitude of reactions such as the oxidation-reduction. Quinones are one product of these reactions and are synthesized from organic, aromatic compounds [242]. Quinones are not aromatic but conjugated and contain at least one benzene-like ring with redox functionality [243]. Anthraquinones are a subgroup of quinones that participate in redox reactions such as the regulation of hydrogen peroxide [243]. Emodin is an anthraquinone that can be found in rhubarb, aloe vera and fo-ti root, which is also known in China as he-shou-wu [244]. Traditional Chinese medicine used these plants to treat viral and bacterial infections as well as bowel abnormalities. Due to its strong redox function and recently discovered anti-inflammatory properties, emodin has been investigated in the heart. Reports showed that emodin inhibited I/R-induced cardiac damage through improvements in the mitochondrial redox regulation [245,246]. Emodin was also reported to attenuate cardiac dysfunction in left coronary artery ligated mice, in part, by inhibiting NF-κB signaling and subsequent inflammation [247]. However, emodin is a strong metal chelator [248], which suggests that emodin can bind to and inhibit zinc-dependent HDACs. Consistent with this hypothesis, our lab published that emodin inhibited HDACs and increased histone acetylation in cardiac myoblasts [119]. Further unpublished data from our lab suggest that emodin inhibits cardiac myocyte hypertrophy, in part, through HDAC-dependent mechanisms. These observations would suggest an epigenetic function for emodin through HDAC inhibition. Our lab is currently investigating the *in vitro* and *in vivo* epigenetic implications for emodin and emodin-rich foods like rhubarb to delineate their roles in diet-gene interactions. 

#### 4.1.11. Stilbenes 

Stilbenes are a small group of phytochemicals that are derived from the phenyl-propanoid pathway via stilbene synthase [249]. While stilbene concentrations are low in the diet, resveratrol is an exception. Resveratrol is found in wine as well as grapes and berries [250] and has been credited for the “French Paradox”. CVD rates in France are lower than the rest of the world despite their high intake of saturated fats [251]. Studies suggest that resveratrol is cardio-protective [89,250,252,253]. Resveratrol was reported to attenuate cardiac damage in response to myocardial infarction [254,255,256,257,258], pressure overload [259,260,261,262,263] and hypertension [90,264,265,266,267,268]. These reports demonstrated resveratrol inhibited oxidative stress and upregulated AMP-activated protein kinase (AMPK) expression and activity [257]. Other reports have confirmed resveratrol improves AMPK levels in the heart [263]. AMPK senses energy needs and stress in the heart. In response to cardiac remodeling, compensatory mechanisms activate AMPK [269]. AMPK activation has been shown to improve cardiac dysfunction [269]. Thus, resveratrol-mediated activation of AMPK is considered cardio-protective. In addition, resveratrol has been shown to stimulate class III sirtuin HDAC activity. This topic has been thoroughly reviewed [91]. Notably, the class III HDAC, Sirt1 regulates AMPK, which leads to a mechanism by which resveratrol-mediated activation of Sirt1 stimulates AMPK expression and activity [257]. Sirt1 is a deacetylase that has been shown to deacetylate lysine residues on histone tails [92]. Thus, most studies have shown that, unlike the phytochemicals discussed above, resveratrol attenuated diabetic cardiac remodeling concomitant with histone H3K9 deacetylation and changes in gene expression. This would suggest that class III HDAC inhibition has negative consequences in the heart. It should be noted that recent proteomic studies have shown that mitochondrial proteins are hyper-acetylated in failing hearts. Moreover, hyper-acetylation of mitochondrial proteins likely result from down-regulation of class III HDACs, which predominantly localize to the mitochondria [270,271]. While these data support a role for resveratrol in the “French Paradox”, doses of resveratrol used in these studies significantly exceed concentrations found in the diet [250]. Nutraceutical companies, however, have developed supplements for human consumption. These nutraceutical companies may impart benefits since a recent double-blind, randomized control trial demonstrated that patients that received 500 mg resveratrol had reduced histone H3K56 acetylation, increased anti-oxidant activation in peripheral blood mononuclear cells (PBMCs) and reduced body fat [272]. While resveratrol activates class III HDACs, its role with zinc-dependent HDACs remains less well-studied. Resveratrol was shown to inhibit class I, II and IV HDACs in hepatoma cells [273]. This would suggest that resveratrol can stimulate the activity of class III NAD^+^-dependent HDACs and can also inhibit zinc-dependent HDACs. Thus, bioactive food compounds may serve multiple epigenetic roles in the control of human health and disease. 

#### 4.1.12. Other Polyphenols

Turmeric is a yellow-pigmented spice that has been used in several cultures including Indian and Southeast Asian cultures for centuries. Turmeric was traditionally used to treat inflammation and flu-like illnesses [274]. Turmeric is isolated from rhizomes of the plant *Curcuma longa* and contains several phytochemicals known as curcuminoids including the well-studied curcumin [275]. Curcumin is a polyphenol that has several hydroxyl groups and two aromatic phenyl rings with each containing a functional methoxy group [275]. Curcumin has been studied for the treatment of many diseases including cancer, Alzheimer’s disease, rheumatoid arthritis and cardiac disease [276]. In the heart, curcumin has been shown to attenuate free fatty acid-induced injuries [277], I/R-induced injuries [278], chemo-induced cardiotoxicity [279,280], hypertension-induced cardiac remodeling [281], diabetes-induced cardiac injuries [282,283] and trauma-induced cardiac dysfunction [284]. Moreover, reports suggest that curcumin’s cardio-protective effects can be converted to humans [93,94,95,285,286,287]. Of these, curcumin was shown to reduce circulating triglycerides [94,95,287] and improve cholesterol status [94], which are two known risk factors in the development of heart disease. Recently, curcumin was shown to inhibit p300/cAMP response element binding protein (p300/CBP)-mediated GATA4 acetylation through the inhibition of HAT activity [96,288]. GATA4 acetylation by p300/CBP stimulates GATA4 transcriptional activation and promotes pathological cardiac gene expression leading to cardiac hypertrophy [289]. Moreover, adrenergic-agonist-induced cardiac myocyte hypertrophy was attenuated with curcumin treatment concomitantly with GATA4 de-acetylation as well as inhibition of GATA4-DNA binding in hypertensive rats [290]. In addition to its inhibitory actions on HATs, curcumin was shown to act as a pan-HDAC inhibitor targeting zinc-dependent HDACs in cancer [291]. Similar to resveratrol, these data suggest multiple levels of epigenetic regulation for curcumin in regulating diet-gene interactions. These data also highlight curcumin as a promising nutraceutical for CVD and HF. However, continued work on curcumin bioavailability is warranted [292,293]. 

### 4.2. Alkaloids

Dietary alkaloids are widely consumed. Alkaloids are precursor compounds that can be derived from ornithine, lysine, tyrosine, tryptophan, nicotinic acid and purine [294]. For example, berberine is an isoquinoline alkaloid derived from tyrosine that naturally occurs in edible and herbal plants including *Hydrastis canadensis, Coptis chinensis, Berberis aquifolium, Berberis vulgaris* and *Berberis aristata*. Moreover, traditional Indian and Chinese medicines have used berberine-enriched plants for the treatment of viral and bacterial infections [295]. More recently, berberine was shown to attenuate diabetes and improve metabolic function [296,297]. In these studies, berberine improved insulin sensitivity through AMPK activation [296] as well as reduced LDL, total cholesterol, circulating triglycerides and increased HDL in the blood [297]. This is of interest since diabetes and metabolic dysfunction are major risk factors for the development of cardiac disease. In this regard, a bioactive capsule that contained several compounds including berberine hydrochloride was shown to attenuate myocardial fibrosis in diabetic rats. These actions were mediated through the inhibition of TGF-β1/Smad [298]. It should be noted that this capsule contained several phytochemicals and, therefore, the impact for berberine hydrochloride on myocardial fibrosis remains unclear. However, it has been reported that berberine improved cardiac function in hypertensive rats by inhibiting STAT3 binding and promoting STAT5a binding to the promoter region of the relaxin gene. This increased relaxin gene expression and subsequently attenuated cardiac fibrosis [299]. Switching of STAT3 for STAT5a at the relaxin gene promoter is controlled by histone H3 acetylation [300]. This is critical since we found that berberine hydrochloride inhibited class I and II HDAC activity [119]. Combined, these data would suggest that berberine-mediated HDAC inhibition would increase histone H3 acetylation at the relaxin gene promoter to inhibit cardiac fibrosis. Further examination of this hypothesis in the heart would be interesting and would provide epigenetic mechanisms by which berberine regulates gene expression.

Danggui Longhui Wan is an active alkaoloid that has been used for more than 4000 years. Danggui Longhui Wan was the customary treatment for chronic myelocytic leukemia and has had moderate success in leukemic disorders without major side effects [301]. The primary bioactive phytochemical in the medicinal recipe, indirubin, has since been isolated and characterized with several aromatic rings. The role for indirubin in cancer has been extensively reviewed [302]. With regard to the heart, indirubin and its derivatives protect against hyperglycemia-induced cardiac injury, aortic constriction-induced hypertrophy, I/R injury, hyperlipidemia-induced cardiac injury and diabetes-induced cardiomyopathy [97,98,99,100,303,304]. Cardiac protection was shown to be mediated in part through the attenuation of c-Jun-N-terminal kinase (JNK) signaling, caspase-3-directed apoptosis, and NF-ĸB expression [303]. Others have reported that indirubin regulated GSK-3β signaling in order to protect cardiac function [97,98,99,100,304]. These results are interesting since class I HDACs have been shown to regulate GSK-3β signaling [150], JNK phosphorylation [50] and NF-κB activation [131]. Since we reported that indirubin inhibited HDAC activity in cardiac tissue [119], these data would suggest that cardio-protection is mediated, in part, through HDAC-dependent actions. Further investigation is needed to elucidate the epigenetic role for indirubin in diet-gene regulation within the heart.

### 4.3. Isothiocyanates

Many foods contain phytochemicals with one or more sulfur groups and are commonly known as organosulfur compounds. Of these, isothiocyanates have been linked with the attenuation of cancer, diabetes, and CVD. Sulforaphane is an isothiocyanate that is found in cruciferous vegetables like broccoli and cauliflower. Early studies showed that sulforaphane inhibited zinc-dependent HDAC activity and, thus, blocked cancer proliferation and induced cancer cell death [101,102,305,306,307,308]. Furthermore, these studies showed that sulforaphane blocked HDAC activity in the cell culture while rodents and humans fed broccoli sprouts [101,102,305,306,307,308]. In the heart, sulforaphane attenuated chemotherapy-induced cardiotoxicity [309,310], I/R injury [311,312], angiotensin II-induced hypertrophy [313], myoblast apoptosis [314], diabetes-induced cardiomyopathy [315,316] and aortic constriction-induced HF [317]. These studies consistently showed that cardio-protective effects of sulforaphane occurs due to the inhibition of oxidative stress. This likely resulted from Nrf2 upregulation [315,316], which is a transcription factor that regulates genes involved in the oxidative stress response. As previously mentioned, class III HDACs regulate Nrf2 [131,318,319]. In addition to its actions directed at Nrf2 induction, sulforaphane was shown to block oxidative stress-induced AMPK inhibition [315]. AMPK is downstream of Nrf2 and upstream of the class III HDAC, Sirt1 [257]. In addition to its role in the regulation of zinc-dependent HDACs and sirtuins, sulforaphane was also shown to attenuate cardiac hypertrophic gene expression by inhibiting GATA4/6 transcriptional activation. This was likely mediated through the inactivation of the MAPKs [320]. HDAC inhibition has previously been shown to inhibit MAPK activity [50]. HAT inhibition controls GATA4 acetylation and subsequent activation [96,288]. However, no report examined the role for sulforaphane in the HDAC-dependent regulation of CVD or HF. This is interesting considering its historical role as a pan-HDAC inhibitor in cancer. Moreover, sulforaphane has been translated to the bedside, which demonstrates the efficacy for this compound as an HDAC inhibitor [307,308]. Combined, these studies suggest further investigation of sulforaphane as an epigenetic regulator of gene expression and cardiac function. Similar to curcumin and resveratrol, sulforaphane likely regulates many epigenetic pathways in the control of human health and disease and these studies should be performed. Lastly, other isothiocyanates including phenethyl isothiocyanate (PEITC) should be investigated in the heart as preliminary evidence, which suggests a cardio-protective role for PEITC [321] as well as a potential role for PEITC as an HDAC inhibitor [322,323].

### 4.4. Other Food Bioactives

Butyrate is a short-chain fatty acid that is metabolized from bacteria within the large intestine and is a well-known short-chain fatty acid HDAC inhibitor [103]. Recent data suggests that gut bacteria play an important role in biological function that governs human health and disease [104]. For example, these bacteria or gut microbiota synthesize butyrate from consumed fibrous, plant-based foods and, once synthesized, butyrate has been shown to inhibit cancer [103], diabetes [324], and CVD [325]. While no epidemiological studies were found linking butyrate to heart health, there is no doubt that consuming fruits, vegetables, and other fibrous, plant-based foods is cardio-protective. Moreover, experimental studies have shown that butyrate is cardio-protective. These studies demonstrated that butyrate protects the heart in an HDAC-dependent manner [326,327]. Butyrate was shown to improve cardiac function through HDAC inhibition in diabetic mice [326]. Moreover, GLUT1 and GLUT4 were upregulated via GLUT1 acetylation and p38 phosphorylation, which leads to improvements in glucose uptake [326]. Similarly, butyrate improved serum cholesterol and left ventricle function via HDAC inhibition in diabetic mice [327]. Like butyrate, valproic acid has been shown to improve cardiac function by acting as an HDAC inhibitor [328]. Since valproic acid is currently approved for the treatment of epilepsy, these data would suggest that short-chain fatty acid HDAC inhibitors are safe and tolerated in humans. Therefore, investigation of HDAC activity in the PBMCs of patients treated with short-chain fatty acids would be of interest. However, it should be cautioned that milli-molar doses of short-chain fatty acids are required for HDAC inhibition and, thus, these compounds likely elicit off-target actions that may contraindicate their therapeutic use for treating CVD/HF.

### 4.5. Whole Foods

Much of this review has focused on individual bioactive food compounds in regulating heart disease. However, phytochemicals are packaged in combination within fruits and vegetables. As a result, it is imperative that we understand how phytochemicals within whole foods solicit epigenetic changes to regulate human health and prevent cardiac disease. It has been reported that grape powder extract improved blood lipid profiles in mice. Improvements in blood lipids occurred, in part, by inhibiting HDACs 2 and 3. This led to peroxisome proliferator-activated receptor alpha (PPARα) gene expression. PPARα regulates hepatic lipid metabolism [241]. Thus, consumption of procyanidin-rich grapes, grape juice, or wine has the potential to elicit epigenetic changes in a manner consistent with heart health [329]. Similarly, foods such as cereals enriched with flavanoids and phenolic acids has been inversely correlated to mortality from coronary heart disease and heart attacks [111]. It remains unclear if the protective actions for fortified cereals on heart disease were mediated through the HDAC inhibition. Considerable work is still needed to understand the epigenetic impact for whole foods on cardiac health. 

## 5. Conclusions

**Figure 2:**
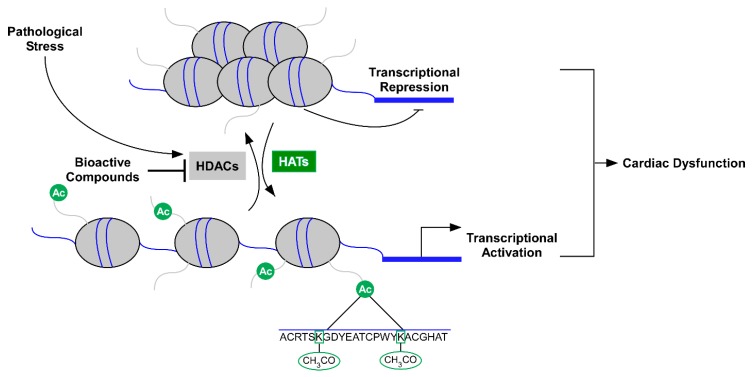
Model demonstrating that food bioactives (phytochemicals) inhibit histone deacetylase (HDAC) activity as a cardio-protective mechanism. HDACs catalyze the removal of acetyl groups from lysine residues on histone tails. Deacetylation of histones leads to changes in electrostatic interactions between DNA and histone proteins that lead to chromatin condensation and gene repression. Conversely, histone acetyl transferases (HATs) add acetyl marks contributing to relaxed chromatin and gene expression. Increased HDAC activity is linked to cardiac dysfunction while inhibition of HDACs is cardio-protective. Thus, food bioactive HDAC inhibitors promote heart health via epigenetic regulation of gene expression.

In this review, we discussed the role for HDAC inhibitors as potential therapeutics for the treatment of HF (Figure 2). In addition, we highlighted food bioactive HDAC inhibitors and discussed their potential implications for the prevention and/or treatment of CVD and HF (Figure 2). The role for diet-gene interactions in human health and disease has been studied extensively over the last couple of decades. Yet recent technologies have improved our understanding for food bio-actives as epigenetic regulators of gene expression [330,331,332]. This diet-epigenetic-gene interaction (nutri-epigenetics) has yielded new and significant insight in the field of nutrition.

The majority of the reports described in this review studied individual dietary compounds in the control of cardiac disease. However, our diet is composed of a plethora of macro-nutrients and micro-nutrients that potentially act in a competitive, additive, or synergistic manner to control cellular function. It has been surmised that the increased intake of fruits, vegetables, and whole grains is beneficial for human health because of the multitude of interactions between macro-molecules and micro-molecules in the regulation of cell function. This can be seen in studies that examine combined food bioactive interactions in various disease models. For example, combination treatment with luteolin and fisetin ameliorated NF-κB signaling and subsequent inflammation in the treatment of hyperglycemia [333]. In addition, food freshness and food preparation, e.g., steaming vs. raw can impact nutrient content and composition as well as phytochemical properties. Thus, it is imperative that future studies investigate the role for food freshness and preparation on macro-molecule and micro-molecule concentration and whether this impacts cellular function. Lastly, future studies examining macro-molecule and micro-molecule interactions on a cellular function will be important for future studies in order to expand our overall understanding within the nutrition field. 

While a major extent of this review focuses on the protective effects of phytochemicals in the heart, it should be noted that over-consumption can contribute to adverse effects. For example, a recent randomized, placebo-controlled crossover trial that supplemented healthy participants on a high-fat diet with curcumin and resveratrol found that serum triglycerides were elevated six hours postprandial [334]. This is consistent with other reports that demonstrated a significant increase in serum triglycerides and total cholesterol in diabetic patients that received resveratrol [335]. Additional reports in humans have associated gastrointestinal/abdominal distress with resveratrol doses at or above 500 mg [336]. However, this may be on an individual basis since this dose and higher have been well-tolerated [335]. Curcumin also has reported adverse effects at high-doses (500–12,000 mg) that include diarrhea, headache, rash, and yellow stool [337]. Phytochemical dose-response and dose-dependency experiments (e.g., IC50 and LD50) in different pathological models are currently underway to alleviate such concerns. However, despite the established safety of many phytochemicals, negative side effects may exist. As such, studies examining safety and efficacy are equally as important as studies elucidating phytochemical benefits. 

Current FDA approved HDAC inhibitors have been developed for the treatment of T-cell lymphoma [26,27,28,29]. Additional HDAC inhibitors are undergoing the long and strenuous process of phase 1-3 trials needed for FDA approval, but none are currently meant for the treatment of HF. The Dietary Supplement Health and Education Act of 1994 (DSHEA) allows for lenient IRB and FDA approval of food-derived substances and phytochemicals [338]. Phytochemicals/nutraceuticals, therefore, can more-readily see human trials compared to current HDAC inhibitors. Several phytochemical nutraceuticals, e.g., curcumin, resveratrol, and sulforaphane have been shown to modulate histone acetylation in human PBMCs, which is described above. These results do not suggest, however, that systemic acetyl-histone modification provide direct mechanisms for human cardio-protection or health. What these results do suggest is that these phytochemical nutraceuticals or their metabolites are capable of inhibiting HDAC activity in the blood. This is important because many phytochemicals that are efficacious in vitro and in vivo are not absorbed or bioavailable. Curcumin, resveratrol, sulforaphane, and other identified phytochemical HDAC inhibitors such as butyrate require further investigation in human subjects but show promise. It would be particularly interesting to supplement foods and nutraceuticals containing these compounds in CVD-susceptible human subjects and examine classic circulating CVD markers such as the natriuretic peptides atrial natriuretic peptide (ANP) and brain natriuretic peptide (BNP). In addition, non-invasive examination of blood pressure as well as cardiac wall-thickness and function via echocardiography would provide useful insight for phytochemical therapeutics in CVD/HF patients. 

In conclusion, food bioactive HDAC inhibitors act as epigenetic regulators of chromatin structure and gene expression. This leads to diet-genome interactions that appear to promote human health and deter cardiac disease. Research investigating food bioactive HDAC inhibitors in the heart is ongoing and will likely yield novel insights within the field of nutritional epigenomics. While this review focused on the role for food bioactive HDAC inhibitors in the heart, it would be naïve to believe that these molecules only target proteins involved in acetylation/deacetylation. This is evident with sulforaphane, which is a molecule that inhibited zinc-dependent HDACs [101,102,305,306,307,308] and activated sirtuins [131,315,316,318,319]. Sulforaphane has also been shown to regulate DNA methylation in order to control gene expression [339,340,341]. Thus, our understanding of food bioactive epigenetic modifiers in health and disease is in its infancy. Lastly, diet-microbiome interactions are likely to yield metabolites that also impact the epigenome. This diet-microbiome-epigenome axis likely plays a critical role in human health. Future studies are likely to explore this relationship, which is currently happening in the gut and brain [341,342,343]. 

## Figures and Tables

**Table 1:** Examples of dietary compounds that regulate histone acetylation.

Phytochemical Class	Compound	Dietary Source	Acetyl Modification	References
Hydroxycinnamic acid	Caffeic acid	Coffee, potatoes, sunflower seeds, skin, of ripened fruit (e.g., berries)	↑ Class III HDAC (Sirts 1 & 3) activity, ↓ class I, IIa & IIb HDAC activity	[72,73]
Hydroxybenzoic acid	Gallic acid	Black tea, berries (e.g., strawberries and blackberries)	↓ Class IIa & IIb HDAC activity	[74]
Flavonol	Quercetin	Teas, peppers, wines, onions, berries, apples	↓ Class I, IIa & IIb HDAC activity, ↑ class III HDAC (Sirt1)	[73,75,76,77]
	Kaempferol	Teas, tomatoes, hops, grapes, grapefruit, strawberries, broccoli, honey, apples, beans	↓ Class I, IIa, IIb & IV HDAC activity, ↑ H3 acetylation; dock HDACs 2, 4, 7 & 8	[73,78,79]
	Myricitrin/Myricetin	Bayberry tree components, wine, berries, vegetables	↓ class I, IIa & IIb HDAC activity	[73]
Flavone	Apigenin	Citrus, onions, celery, chamomile tea	↓ Class I, IIa & IIb HDAC activity, ↑ H3 acetylation; dock class I HDACs	[73,80,81,82]
	Luteolin	Celery, parsley, broccoli, onions, carrots, peppers, cabbages, apples	↓ class I, IIa & IIb HDAC activity, ↑ H3 acetylation, dock class I HDACs	[73,82]
	Baicalein/Baicalin	*Scutellaria baicalensis*	↓ class I, IIa & IIb HDAC activity, ↓ HDACs 1, 4 & 5 expression	[73,83,84,85]
Flavanol (catechin)	EGCG	Green tea, black tea, apples, berries, chocolate	↓ class I, IIa & IIb HDAC activity, ↑ H3 acetylation	[73,86,87]
Flavanolol	Dihydromyricetin	*Ampelopsis grossedentata* leaves and stems	↓ Class I, IIa & IIb HDAC activity	[73]
Proanthocyanidin	Grape Seed	Grapes	↓ HDAC2 & HDAC3 activity, ↑ Histone acetylation	[88]
Quinone	Emodin	Rhubarb, aloe vera, buckthorn, knotweed, fo-ti root	↓ class I, IIa & IIb HDAC activity, ↑ H3 acetylation	[73]
Stilbene	Resveratrol	Wine, grapes, berries	↑ Sirt1, ↓ H3 acetylation, ↓class I, II & IV HDACs	[89,90,91,92]
Curcuminoid	Curcumin	Tumeric	↓ HAT activity, ↓ HDAC activity, ↓ class I, IIa, IIb & IV HDAC expression	[93,94,95,96]
Alkaloid	Berberine Hydrochloride	*Hydrastis canadensis, Coptis chinensis, Berberis aquifolium, Berberis vulgaris, Berberis aristata*	↓ Class I & IIb HDAC activity	[73]
	Indirubin	Glastum, buckwheat	↓ Class I & IIb HDAC activity	[73]
Isothiocyanate	Sulforaphane	Cruciferous vegetables (e.g., broccoli and cauliflower)	↓ Class I, IIa & IIb HDAC activity	[97,98,99,100,101,102]
Short-chain fatty acid	Butyrate	Bacterial metabolism of fibrous foods	↓ HDAC activity, ↓ HDAC4	[103,104]

↑ Increased; ↓ Decreased.
